# Reduction of Mature B Cells and Immunoglobulins Results in Increased Trabecular Bone

**DOI:** 10.1002/jbm4.10670

**Published:** 2022-08-30

**Authors:** Marie K. Lagerquist, Priti Gupta, Edina Sehic, Karin L. Horkeby, Julia M. Scheffler, Jauquline Nordqvist, Lina Lawenius, Ulrika Islander, Carmen Corciulo, Petra Henning, Hans Carlsten, Cecilia Engdahl

**Affiliations:** ^1^ Sahlgrenska Osteoporosis Centre, Centre for Bone and Arthritis Research, Department of Internal Medicine and Clinical Nutrition, Institute of Medicine, Sahlgrenska Academy University of Gothenburg Gothenburg Sweden; ^2^ Sahlgrenska Osteoporosis Centre, Centre for Bone and Arthritis Research, Department of Rheumatology and Inflammation Research, Institute of Medicine, Sahlgrenska Academy University of Gothenburg Gothenburg Sweden

**Keywords:** B LYMPHOCYTES, IMMUNOGLOBULIN, OSTEOCLASTS, OSTEOIMMUNOLOGY, TRABECULAR BONE

## Abstract

Inflammation has a significant effect on bone remodeling and can result in bone loss via increased stimulation of osteoclasts. Activated immunoglobulins, especially autoantibodies, can increase osteoclastogenesis and are associated with pathological bone loss. Whether immunoglobulins and mature B lymphocytes are important for general bone architecture has not been completely determined. Here we demonstrate, using a transgenic mouse model, that reduction of mature B cells and immunoglobulins leads to increased trabecular bone mass compared to wild‐type (WT) littermate controls. This bone effect is associated with a decrease in the number of osteoclasts and reduced bone resorption, despite decreased expression of osteoprotegerin. We also demonstrate that the reduction of mature B cells and immunoglobulins do not prevent bone loss caused by estrogen deficiency or arthritis compared to WT littermate controls. In conclusion, the reduction of mature B cells and immunoglobulins results in disturbed regulation of trabecular bone turnover in healthy conditions but is dispensable for pathological bone loss. © 2022 The Authors. *JBMR Plus* published by Wiley Periodicals LLC on behalf of American Society for Bone and Mineral Research.

## Introduction

The immune and bone systems share numerous regulatory factors including receptors and signaling molecules. Bone homeostasis is maintained by a coordinated action of bone‐forming osteoblasts and bone‐resorbing osteoclasts. This balance can be disturbed by immune activation, resulting in the pathophysiological immune‐mediated bone loss that can be seen in some autoimmune diseases. Osteoclasts are derived from monocyte/macrophage precursors of the hematopoietic lineage, and they differentiate into multinucleated osteoclasts under the influence of macrophage colony‐stimulating factor (M‐CSF) and the receptor activator of nuclear factor κB ligand (RANKL). Several pro‐inflammatory cytokines such as interleukin‐17 (IL‐17), IL‐6, and tumor necrosis factor‐alpha (TNFα) can further increase osteoclastogenesis.

B lymphocytes (B cells) have a close and multifaceted relationship with bone. Memory B cells need support from osteoblasts in bone marrow niches for survival. B cells can also influence bone by producing RANKL as well as by being one of the major contributors of osteoprotegerin (OPG), a decoy receptor for RANKL, that alters the differentiation and activation of osteoclasts.^(^ ^1^, ^2^ ^)^ There is also evidence showing that a subset of early immature B cells can develop into osteoclasts after stimulation with M‐CSF and RANKL *in vitro*.^(^ ^3^ ^)^ Mature B cells and plasma cells are responsible for the generation of immunoglobulins and antibodies, which are secretory products of the adaptive immune system. The major class of immunoglobulins found in serum is immunoglobulin G (IgG) and activated IgGs regulate immunogenic and tolerogenic responses via binding to fragment crystallizable gamma receptors (FcγRs), which are expressed in all hematopoietic cells, including osteoclasts.^(^ ^4^, ^5^ ^)^ The interaction between activated IgGs and bone has primarily been shown in rheumatoid arthritis (RA). RA patients who express autoantibodies against citrullinated proteins (ACPAs) have a lower bone mineral density (BMD) and this bone loss can be seen before established disease in pre‐RA patients^(^ ^6^, ^7^ ^)^ as well as in patients with active RA.^(^ ^8^, ^9^ ^)^ We and others have shown that heat‐activated IgGs can enhance RANKL‐mediated osteoclastogenesis in *in vitro* cultures of both human and murine cells as well as induce local bone loss *in vivo* in mice.^(^ ^4^, ^5^, ^10^, ^11^, ^12^ ^)^ In addition, Zeng and colleagues^(^ ^13^ ^)^ have shown that activated IgGs can stimulate osteoclastogenesis independent of RANKL and other inflammatory cytokines.

Several studies from us and others have found that the presence of activated IgGs can stimulate bone loss.^(^ ^4^, ^5^, ^10^, ^11^, ^12^, ^14^, ^15^ ^)^ However, there are no studies investigating the impact of IgG deficiency on bone because there are no animal models or methods available to completely remove only IgGs. The B10.129S2(B6)‐lghm^tm1Cgn^/J (muMT) mice lack immunoglobulins, including IgGs, and mature B cells, including plasma cells, and can be used as a model to determine the importance of reduction in immunoglobulins and mature B cells. The muMT mice have a knockout mutation of the gene encoding the heavy chain of immunoglobulin M (IgM), which forces progenitor B cells into apoptosis before further development.^(^ ^16^, ^17^ ^)^ The few studies investigating the skeleton in muMT mice have used separately bred wild‐type (WT) mice as controls and they have demonstrated conflicting findings. Horowitz and colleagues^(^ ^18^ ^)^ show no skeletal differences between muMT mice and separately bred controls. In contrast, Li and colleagues^(^ ^19^ ^)^ and Khass and colleagues^(^ ^20^ ^)^ show a reduction in both cortical and trabecular BMD in muMT mice compared to separately bred WT controls, and this phenotype can be rescued after B cell reconstitution by adoptive transfer.

The role of B cells and immunoglobulins in ovariectomy (ovx)‐induced bone loss is unclear. The number of B cells, mainly the immature B cell population, increases following estrogen deficiency, but the bone loss after ovx is similar in mice that lack most of their B cells and WT mice.^(^ ^21^, ^22^ ^)^ Furthermore, muMT mice, which lack mature B cells, display the same degree of ovx‐induced bone loss compared to separately bred WT mice.^(^ ^23^ ^)^ These studies indicate that B cells are not required for ovx‐induced bone loss. In contrast, Onal and colleagues^(^ ^24^ ^)^ have shown that ovx‐induced bone loss is partly dependent on RANKL expression in B cells. It has been shown that the level of immunoglobulins is affected by estrogen status,^(^ ^25^ ^)^ but their direct involvement in ovx‐induced bone loss is not completely understood.

B cells are shown to be important in the development of collagen‐induced arthritis (CIA), an animal model of RA, and the development of CIA is abrogated in muMT mice.^(^ ^26^ ^)^ CIA induction can also be blocked by B cell depletion in WT mice.^(^ ^27^ ^)^ Mice deficient in expression of functional FcγRs, and thereby lacking actions of IgGs, are protected against inflammatory arthritis, demonstrating the involvement of activated antibodies in the development of arthritis.^(^ ^5^, ^28^, ^29^ ^)^ The development of antigen‐induced arthritis (AIA), a model of monoarthritis, is not dependent on B cells and develops normally in muMT mice.^(^ ^30^ ^)^ Using this model, we have demonstrated^(^ ^10^, ^14^ ^)^ that murine ACPAs, but not heat‐activated murine IgGs, influence arthritis‐induced bone loss, but the exact role of mature B cells and immunoglobulins for this arthritis‐induced bone loss is still unclear.

We hypothesized that mature B cells and immunoglobulins are involved in osteoclast activation and regulation of bone architecture in both healthy and pathogenic conditions. To test this concept, we investigated the bone in detail in muMT mice lacking immunoglobulins and mature B cells and compared them to WT littermate controls. We provide novel data showing that healthy muMT mice display increased trabecular bone density and a reduced number of osteoclasts compared to littermate WT controls. We also show that the reduction of mature B cells and immunoglobulins has limited importance for the pathogenic bone loss investigated in this study.

## Materials and Methods

### Generation of mice

muMT mice, which have a deletion of the IgM heavy chain gene (The Jackson Laboratory, Bar Harbor, ME, USA; JAX stock #002249)^(^ ^16^ ^)^ and thereby lack mature B cells and immunoglobulins, were backcrossed more than five generations on C57BL/6J background (Taconic Biosciences, Ejby, Denmark), and homozygous muMT and WT littermates were used in the experiments. The following primer pairs were used for genotyping: WT forward primer (5′‐GAA GAG GAC GAT GAA GGT GG‐3′), muMT forward primer (5′‐TTG TGC CCA GTC ATA GCC GAAT‐3′), and a common reverse primer (5′‐CCG TCT AGC TTG AGC TAT TAG G‐3′). The mice were housed at the Sahlgrenska Academy, Laboratory of Experimental Biomedicine in Gothenburg, Sweden, and fed a phytoestrogen‐free pellet diet (Rodent diet Harlan 2016) *ad libitum*. The study was approved by the animal ethics committee in Gothenburg (1‐2017). Female mice were terminated at different time points: 6 weeks (prepubertal), 12 weeks (postpubertal/young adult), and 16 weeks (adult after the peak growth phase). Male mice were euthanized at 16 weeks of age.

### Reconstitution of activated immunoglobulins in muMT mice

Polyclonal IgG was isolated from pooled serum of intact C57/BL6J mice of different ages and sexes using a protein G spin column (GE Healthcare, Sigma‐Aldrich, Sollentuna, Sweden) according to the manufacturer's instructions. Protein concentration was determined using Mouse IgG enzyme‐linked immunosorbent assay (ELISA) Quantitation set (Bethyl Laboratories, Nordic BioSite, Taby, Sweden) and detergent compatible (DC) protein assay (Bio‐Rad Laboratories AB, Solna, Sweden) according to the manufacturer's instructions. Activated polyclonal IgG complexes were obtained by heat aggregation to form complexes at 63°C for 30 minutes. This allows the IgGs to interact with FcγRs in the absence of antigen binding.^(^ ^4^, ^11^ ^)^ Then, 0.03 mL of polyclonal heat‐activated IgGs (7.5 mg/kg), diluted in phosphate‐buffered saline (PBS), were injected into the knee joint of female muMT mice at 11 and 12 weeks of age and compared to the contralateral leg with an intraarticular injection with PBS. Female WT littermates with an intraarticular injection with PBS were used for comparison. The experiment was terminated at 13 weeks of age.

### Ovx‐induced bone loss

Ovx and sham‐operations were performed in 16‐week‐old female muMT mice and WT littermates as described.^(^ ^31^ ^)^ Surgery was performed under anesthesia with isoflurane (Baxter Medical AB, Kista, Sweden), and carprofen (Rimadyl; Orion Pharma Ab. Animal Health, Sollentuna, Sweden) was given preoperatively as an analgesic. Surgery efficiency was confirmed by weighing the uterus at termination 4 weeks after the operation. The data from two independent ovx and sham‐operation experiments were pooled.

### Arthritis‐induced bone loss

The antigen‐induced arthritis (AIA) model, previously used for analyzing arthritis‐induced bone loss,^(^ ^32^ ^)^ was induced in 11‐week‐old male muMT and WT littermate controls. The mice were immunized by injecting 0.1 mL of 0.1 mg/mL murine bovine serum albumin (mBSA; Sigma‐Aldrich) emulsified in equal amounts of complete Freund's adjuvant (CFA) (Sigma‐Aldrich) containing 1 mg/mL heat‐inactivated *Mycobacterium tuberculosis* in the base of the tail. This primary systemic immunization was followed by a local intraarticular 0.03‐mL injection of mBSA in one knee (arthritic knee) and PBS in the contralateral knee (non‐arthritic knee) after 1 week. The PBS‐injected side was used as internal control, as described.^(^ ^32^ ^)^ The local arthritis inflammation induction was monitored until termination, 2 weeks after the primary immunization, and the diameter of the knee was measured using a caliper.

### Cell preparation and flow cytometry

Bone marrow cells were flushed out and harvested from the femoral bone by using a syringe with PBS. Splenocytes were isolated and a single‐cell suspension in PBS was obtained by passing them through a cell strainer. Pelleted cells were resuspended in Tris‐buffer containing 0.83% NH_4_Cl solution to lyse erythrocytes and then washed in PBS. The total number of leukocytes was counted using a cell counter (Sysmex, Europe GmBH, Norderstedt, Germany). The following fluorochrome conjugated anti‐mouse antibodies were used: allophycocyanin (APC)‐conjugated anti‐CD267 (TACI), APC‐conjugated anti‐CD117 (cKit), and phycoerythrin (PE)‐conjugated anti‐Gr1 (eBioscience, Thermo Fisher Scientific, Gothenburg, Sweden); PE‐conjugated anti‐CD19, APC‐cyaninine7 (Cy7)‐conjugated anti‐CD3, Brilliant Violet 421‐conjugated anti‐CD138, PE‐conjugated anti‐IgM, and V450‐conjugated anti‐CD11b (Becton Dickinson and company, Franklin, NJ, USA); and PE‐Cy7‐conjugated anti‐B220, APC‐conjugated anti‐CD4 and MCSF‐R, PE‐Cy7‐conjugated anti‐CD8, fluorescein isothiocyanate (FITC)‐conjugated F4/80, and PE‐conjugated RANK (BioLegend, Nordic BioSite). Analyses were performed using FACSVerse (Becton Dickinson) and FlowJo version 10 (FlowJo 10.6.2) (see gating strategies in Figs. S1 and S2). All frequencies of cells were based on either CD3^−^ cells (B cells, plasma cells, monocytes, pre‐osteoclasts, and neutrophils) or B220^−^ cells (T cells and T helper cells).

### Enzyme‐linked immunosorbent spot assay

Quantification of IgG, immunoglobulin A (IgA), and IgM secretion from bone marrow and spleen cells of 16‐week‐old female mice was performed using the enzyme‐linked immunosorbent spot (Elispot) technique. 96‐well nitrocellulose plates (Millipore, Thermo Fisher Scientific) were coated with affinity‐purified F(ab′)2 fragments of goat‐anti mouse IgG, IgA, and IgM. Cell suspensions of isolated bone marrow and spleen were added and incubated at 37°C with 5% CO_2_ for 3.5 hours. Alkaline phosphatase‐conjugated goat anti‐mouse IgG, IgA, and IgM antibodies were added to detect antibody‐producing cells and finally incubated with 5‐bromo‐4‐chloro‐3‐indolyl phosphate and nitro‐blue tetrazolium for visualization of the spots using a microscope.

### Murine serum analyses

IgG, IgM, and IgA serum levels were measured using commercially available ELISA kits (Bethyl Laboratories, Nordic BioSite). Levels below the assay range were set to a level that was the lowest detectable level according to the kit.

As a marker for bone resorption, serum levels of C‐terminal type I collagen fragments (CTX‐I) were determined with a commercially available ELISA Crosslaps kit (Immunodiagnostic Systems, Boldon, UK). As a marker of bone formation, serum levels of N‐terminal propeptide of type I procollagen (P1NP) were assessed with a commercially available EIA P1NP kit (Immunodiagnostic Systems).

Levels of mBSA‐specific antibodies were assessed in serum of AIA mice using an in‐house ELISA, as described.^(^ ^33^ ^)^ Briefly, ELISA plates were coated with 0.01 mg/mL mBSA, blocked and washed with casein (Sigma‐Aldrich) before adding the serum. Bound anti‐mBSA antibodies were detected by horseradish peroxidase (HRP)‐conjugated rabbit anti‐mouse IgG (DAKO, Agilent, Santa Clara, CA USA).

### X‐ray analyses

Dual‐energy X‐ray absorptiometry (DXA) analyses were performed to assess total body areal bone mineral density (aBMD), lumbar spine (L_3_–L_6_) aBMD, and lean mass in 5‐week‐old, 12‐week‐old, and 16‐week‐old female and male mice, using Faxitron UltraFocus DXA of 40 kV and 0.28 mA for 2.53 seconds, with the spatial resolution of 24 μm using 2X geometric magnification (Faxitron Bioptics, LLC, Tucson, AZ, USA). Total body aBMD and lumbar spine aBMD were also investigated before and after ovx and sham operations. Arthritis‐induced bone loss was investigated by analyzing tibia epiphyseal aBMD after AIA induction, as described.^(^ ^10^ ^)^

Tibia length was measured using a caliper before peripheral quantitative computed tomography (pQCT) analyses. Trabecular and cortical bone in the tibia were analyzed using the Stratec pQCT XCT Research M (software version 5.4; Norland, Fort Atkinson, WI, USA) at the resolution of 70 μm. The trabecular bone region was defined in the metaphysis at 0.4 mm from the proximal growth plate in the distal direction by setting an inner area to 45% of the total cross‐sectional area. Cortical thickness was determined by analyzing scans of the mid‐diaphyseal region of the tibia.

High‐resolution microcomputed tomography (μCT) was performed in the tibia and vertebrae (L_5_) using a Skyscan 1172 scanner (Bruker MicroCT, Aartselaar, Belgium) as described.^(^ ^34^ ^)^ The trabecular bone of the tibia was investigated distal to the growth plate in the metaphysis of 6‐week‐old and 16‐week‐old female mice, 16‐week‐old male mice, after intraarticular injection with activated polyclonal IgG and after AIA induction. In addition, trabecular bone in the lumbar spine (vertebrae L_5_) was investigated in 6‐week‐old and 16‐week‐old female mice. The cortical μCT measurements in the tibia were performed in the mid‐diaphyseal region.

### Histological examination

Bone tissues were fixed in 4% formaldehyde, decalcified in 10% EDTA, paraffin‐embedded, and cut into 20‐μm thick sections (sectioning was performed at Histocenter, Mölndal, Sweden). The knee sections from AIA mice were stained with hematoxylin and eosin. For osteoclast staining, sections were stained for tartrate resistant acid phosphatase (TRAP) using a Leukocyte Acid Phosphate kit (Sigma‐Aldrich). Synovitis, bone erosion, and cartilage damage were graded in a blinded manner (by researchers CE and ES) using a blunt three‐grade histological scoring system (mild, moderate, or severe) as described by Liphardt and colleagues.^(^ ^33^ ^)^

Osteoclast number and osteoclast surface attached to the bone were quantified in the epiphyseal and metaphyseal parts of the tibia in the knee sections from the AIA experiment, and both osteoclast and osteoblast number and surface attached to the bone were quantified in the metaphyseal part of the femur in the 6‐week‐old and 16‐week‐old female mice. Osteoclasts were defined as TRAP‐positive cells with three or more nuclei and cuboidal cells at the bone surface were considered as osteoblasts. All histological analyses were performed using a microscope (Nikon, Tokyo, Japan) and the image analysis system Osteomeasure (OsteoMetrics, Decatur, GA, USA). All histological assessments were performed in a blinded manner.

### Osteoclast ex vivo differentiation

Murine osteoclast differentiation was studied *ex vivo* by RANKL‐induced differentiation of crude bone marrow cells (BMCs) or bone marrow macrophages (BMMs) isolated from 3‐month‐old to 4‐month‐old muMT and WT mice.

BMCs were isolated by flushing bone marrow from femoral and tibial bones with α‐MEM medium (Invitrogen, Carlsbad, CA, USA) using a syringe and 30G needle. BMCs were spot seeded at a density of 750000/20 μL cells per well in the center of a 48‐well plate and cultivated in complete α‐MEM medium containing 10% fetal bovine serum (FBS) (Sigma‐Aldrich, 12103C‐500ML), 50 μg/mL gentamicin (Gibco, Thermo Fisher Scientific15750060), 1% penicillin/streptomycin (Gibco, Thermo Fisher Scientific 15140122), and 2mM GlutaMAX (Gibco, Thermo Fisher Scientific; 35050061), at 37°C overnight. The following day, the medium was replaced with 30 ng/mL M‐CSF (R&D Systems, Barton, UK 416‐ML‐050) and 4 ng/mL RANKL (R&D Systems; 462‐TEC) (MRL; M‐CSF + RANKL treatment). The culture medium was changed after 3 days, and larger polynucleated cells were observed after 7 days.

BMMs were cultivated in complete α‐MEM medium with 30 ng/mL M‐CSF (R&D Systems; 416‐ML‐050) in suspension culture dishes (Corning, 430591, Thermo Fisher Scientific), at 37°C.^(^ ^35^ ^)^ After 2 days, nonadherent cells were washed away with PBS and 0.02% EDTA in PBS was used to detach the adherent BMMs from the culture dish. BMMs were spot seeded in the center of the wells at a density of 5000/5 μL cells in 96‐well plates and incubated with 30 ng/mL M‐CSF alone or in combination with 4 ng/mL RANKL (MRL) to induce osteoclast differentiation. The medium was changed after 3 days, a time point at which the cells were mononucleated or binucleated preosteoclasts, and after an additional 2–3 days, larger multinucleated cells were observed.

The formation of osteoclasts was evaluated by TRAP staining (Sigma‐Aldrich; 386A‐1KT) according to the manufacturer's instructions. The number of TRAP‐positive cells containing three or more nuclei was counted and defined as an osteoclast using a microscope (Nikon) and an image analysis system by two independent investigators (OsteoMeasure; OsteoMetrics). The osteoclast surface area measurement was performed using FIJI open‐source image processing package (https://imagej.net/Fiji/Downloads). Pictures were converted to an 8‐bit image for analysis. To trace the cells the “polygonal and freehand selection tool” was used, and the averaged surface areas in muMT compared to the WT were calculated.

### Osteoblast formation

Bone marrow was removed from the diaphysis of the femoral and tibial bones of 3‐month‐old muMT and WT littermate mice. The diaphyseal bone was incubated in collagenase II (2 mg/mL) solution at 37°C for 2 hours followed by a wash with PBS for removing non‐bone cells. The diaphyseal bone was cut into small pieces (1–2 mm) and transferred into 12‐well plates containing complete α‐MEM including 100 U/mL penicillin, 50 μg/mL streptomycin, 50 μg/mL gentamicin, 1.25 μg/mL amphotericin B, 100 μg/mL ascorbate, and 10% FBS. The medium was changed after 2 days, followed by medium change every 3 days during cultivation. Bone cells started to migrate from the bone after 3–5 days and after 10 days the osteoblastic cells were lysed for RNA extraction.

### RNA isolation and quantitative real‐time PCR analysis

Both ends of the tibias were cut and BM was spun out into a tube that was snap‐frozen in liquid nitrogen. L_6_ vertebral bodies were placed in RNAlater (QIAGEN, Hilden, Germany) and stored at −80°C. Total RNA was isolated from vertebrae and BM using TRIzol reagent (Life Technologies, Thermo Fisher Scientific) followed by the RNeasy Mini Kit (QIAGEN). In addition, RNA was isolated from osteoblasts cultivated as described above using RNeasy Micro Kit (QIAGEN; 74004). The RNA was reverse transcribed into complementary DNA (cDNA) using a High‐Capacity cDNA Reverse Transcription Kit (Thermo Fisher Scientific, 4368814). Quantitative real‐time PCR (qPCR) analyses were performed using predesigned TaqMan Assays and TaqMan Fast Advanced Master Mix (Thermo Fisher Scientific; 4444556). The following predesigned real‐time PCR assays from Applied Biosystems (Thermo Fisher Scientific) were used for gene expression analysis: OPG (*Tnfrsf11b*; Mm0043545_m1), RANK ligand (Rankl) (*Tnfsf11*; Mm00441908_m1), MCSF‐receptor (*Csf1r*; Mm01192931_m1), *IL‐6* (Mm00446190_m1), *IL17a* (Mm00439619_m1), *TNFα* (Mm00443258_m1), *IL23a* (Mm00518984_m1), RAR‐related orphan receptor gamma (*Rorc*; Mm01261019_g1), chemokine C‐X2‐C motif receptor 1 (*Cxcr1* Mm00583730_m1), Nuclear factor of activated T‐cells (Nfatc1; Mm00479445_m), RANK (*Tnfsra11a*; Mm00437135_m1), TRAP (*Acp5*; Mm00475698_m1), Dickkopf (*Dkk1*; Mm00438422_m1), ALP (*Alpl*; Mm00475834_m1), a subunit of nuclear factor κ‐light‐chain‐enhancer of activated B cells (NF‐κB) (*p52*; Mm00479810_g1), Cathepsin K (*Ctsk*; Mm00484036_m), Osteocalcin (*Bglap*; Mm03413826_mH), and *Runx2* (Mm00501580_m1). The housekeeping gene 18S (4310893E) (Thermo Fisher Scientific) was used as endogenous control. Gene expression values were calculated based on the delta‐delta threshold cycle (ΔΔCt) method^(^ ^36^ ^)^ where the expression of the WT mice (control), is set to 1 and the expression of the muMT mice is relative to the control group and shown as fold change values.

### Statistics

All analyses were performed with GraphPad Prism software (Graph Pad Software Inc., La Jolla, CA, USA) or SPSS software 21.0 (IBM Corp., Armonk, NY, USA). Logarithmic transformation of data was performed when needed to increase normal distribution. The statistical difference between two independent groups (WT and muMT) was calculated using two‐sided unpaired Student's *t* test. In the ovx experiment, analysis of variance (ANOVA) followed by Tukey's post hoc test was used. If unequal variance was detected with Levene's test, Dunnett's T3 was used as post hoc test. The ovx data were pooled from two independent experiments, and analysis of covariance (ANCOVA) was used for adjustment of day‐to‐day variance when needed. A two‐sided paired Student's *t* test was used when internal comparisons were performed between the arthritic and nonarthritic sides in the AIA experiment. The nonparametric Mann‐Whitney test was used for ordinal scale comparisons of the histological scoring in the AIA experiment. The interaction factor from a two‐way ANOVA analysis was used to determine if the effect of the intervention (ovx or arthritis) differed between muMT mice and WT littermates. Data on knee swelling is presented in Kaplan‐Meier curves as differences from baseline, and the area under the curve was used to determine differences in disease.

Tables, as well as DXA figures showing data over time, are given as mean ± SE. In other figures all data are plotted as box plots, with median, interquartile range, and maximum and minimum values. A value of *p* < 0.05 was considered significant.

Power analysis using P*Power 3.1 indicated that a minimum of seven mice per group provides 80% power to detect a 1.5‐standard deviation (SD) change from WT mice (fluorescence‐activated cell sorting [FACS], serum and skeletal analyses) and that six mice per group provide 80% power to detect a 1.8‐SD change from WT mice (histology and qPCR analyses). Actual sample sizes are indicated in the figure and table legends.

## Results

### muMT mice display alterations in B cells and immunoglobulins as compared to littermate WT controls

**Fig. 1:**
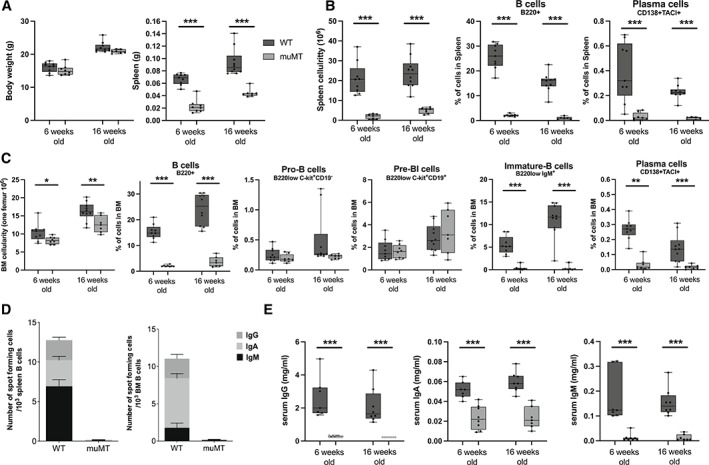
Physiological assessment of muMT mice that lack mature B cells and immunoglobulins compared to WT littermates. (*A*) Body and spleen weights in muMT and WT mice in 6‐week‐old and 16‐week‐old female mice. (*B*) Numbers of splenocytes and frequencies of plasma and B cells in spleen. (*C*) Numbers of BM cells per femur and frequencies of plasma and B cells in BM. (*D*) Immunoglobulin expressing cells in spleen and BM in 16‐week‐old female mice. Presented as interlined box, where IgG, IgA, and IgM expressions are displayed together. (*E*) IgG, IgA, and IgM levels in serum. Student's *t* test was used to assess the differences between WT and muMT mice at each time point. *n* = 6–10. *p < 0.05, **p < 0.01, ***p < 0.001. BM = bone marrow; WT = wild type.

First, we determined the phenotype of muMT mice compared to littermate controls, after backcrossing more than five generations to C57BL/6J background. The total body weight as well as weights of liver, thymus, uterus, and gonadal fat were unaffected in the muMT mice compared to WT littermate mice (Fig. 1*A* and Table S1). Spleen weight was reduced in muMT mice, followed by a dramatic decrease in spleen cellularity (Fig. 1*A*,*B*). There was a clear reduction of the frequency of B cells (B220^+^) and a severely diminished frequency of plasma cells (CD138^+^TACI^+^) (Fig. 1*B*). Also, bone marrow (BM) cellularity was decreased with a decrease in the frequency of all B cells (B220^+^) in muMT mice compared to WT littermates (Fig. 1*C*). Early pro‐B (B220low Ckit^+^ CD19^−^) and pre‐B220low Ckit^+^ CD19^+^ (BI) cell frequencies were unaffected in muMT mice compared to WT littermates, whereas both immature B cells (B220low IgM^+^), as well as plasma cells (CD138^+^TACI^+^) were decreased in BM (Fig. 1*C*). The frequency of T cells, primarily CD4^+^, T‐helper cells, as well as neutrophils and monocytes, were increased in the spleen and BM of muMT mice. However, due to the reduced total cell number, there were no differences in absolute numbers (data not shown). The number of IgG‐, IgA‐, and IgM‐expressing cells in both BM and spleen, as determined with ELISPOT, was severely diminished in muMT mice compared to WT littermates (Fig. 1*D*). IgG, IgA, and IgM levels in serum, determined with ELISA, were below the detection limit in several muMT mice, and significantly reduced compared to WT littermates (Fig. 1*E*).

### Trabecular bone mass is increased in muMT mice

**Fig. 2:**
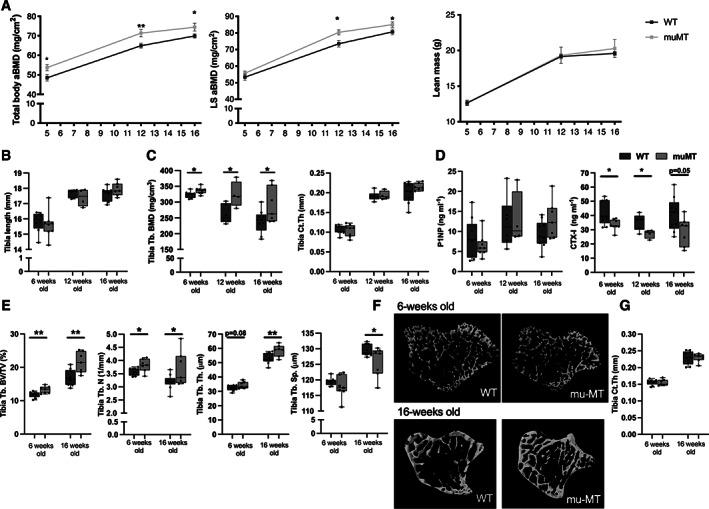
Mice deficient in mature B cells and immunoglobulins show increased trabecular bone. (*A*) Total body aBMD, LS L_3_–L_6_ aBMD, and lean mass measured with DXA in 5‐week‐old, 12‐week‐old, and 16‐week‐old female muMT and WT littermate mice. *Indicates significant differences determined with Student's *t* test. *n* = 7–10. (*B*) Tibia length, (*C*) Tb.BMD, and Ct.Th were analyzed with pQCT in 6‐week‐old, 12‐week‐old, and 16‐week‐old female muMT and WT littermate mice. (*D*) Serum levels of P1NP (bone formation marker) and CTX (bone resorption marker). *n* = 4–9. (*E*) Trabecular BV/TV, Tb.N, Tb.Sp, and Tb.Th analyzed with high‐resolution μCT in 6‐week‐old, and 16‐week‐old female muMT and WT mice. (*F*) Representative images of trabecular bone in muMT and WT littermates. (*G*) Ct.Th was analyzed with μCT. Student's *t* test was used to assess the differences between muMT and WT littermate mice at each time point. *n* = 7–10. *p < 0.05, **p < 0.01. aBMD = areal BMD; BMD = bone mineral density; BV/TV = bone volume per total volume; Ct.Th = cortical thickness; LS = lumbar spine; Tb.BMD = trabecular BMD; Tb.N = trabecular number; Tb.Sp = trabecular separation; Tb.Th = trabecular thickness; WT = wild‐type.

To determine if deficiency of immunoglobulins and mature B cells affects the skeleton, we first analyzed the mice *in vivo* with DXA. Total body aBMD was significantly higher in 5‐week‐old, 12‐week‐old, and 16‐week‐old female muMT mice compared to WT littermates (Fig. 2*A*). Lumbar spine aBMD was significantly higher in 12‐week‐old and 16‐week‐old female muMT mice compared to WT littermates while no difference was seen in 5‐week‐old female mice. No differences were observed in total weight (Fig. 1*A*), lean mass (Fig. 2*A*), or tibia length (Fig. 2*B*) of the mice, indicating that there is no size difference between muMT and WT female mice.

The tibia was further investigated *ex vivo* using pQCT in 6‐week‐old, 12‐week‐old, and 16‐week‐old female mice. Trabecular BMD was higher in muMT mice compared to WT littermates at all time points, whereas no effect was observed in cortical thickness (Fig. 2*C*). Serum markers of bone remodeling showed a significantly lower level of the bone resorption marker CTX‐I in 6‐week‐old and 12‐week‐old muMT mice compared to WT mice, and a strong tendency towards a decrease was also seen in 16‐week‐old female mice, whereas no alteration was observed in the bone formation marker P1NP (Fig. 2*D*).

Trabecular microstructure in tibia was investigated in 6‐week‐old and 16‐week‐old females using μCT and the trabecular bone volume fraction (BV/TV) was higher in muMT than in WT at both time points (Fig. 2*E*,*F*). Analysis of trabecular number, trabecular separation, and trabecular thickness revealed significant differences between muMT and WT mice at 16 weeks of age, and a significant difference in trabecular number, as well as a tendency to increase in trabecular thickness in 6‐week‐old muMT mice compared to WT littermates (Fig. 2*E*). An increase in trabecular bone density was also seen in the axial skeleton, where lumbar spine trabecular BV/TV, measured in vertebrae L_5_, was significantly increased in both 6‐week‐old and 16‐week‐old female muMT mice compared to WT littermates (Fig. S3). No significant alterations in cortical thickness with μCT analyses were detected in 6‐week‐old or 16‐week‐old female (Fig. 2*G*) muMT mice compared to WT littermates.

**Fig. 3:**
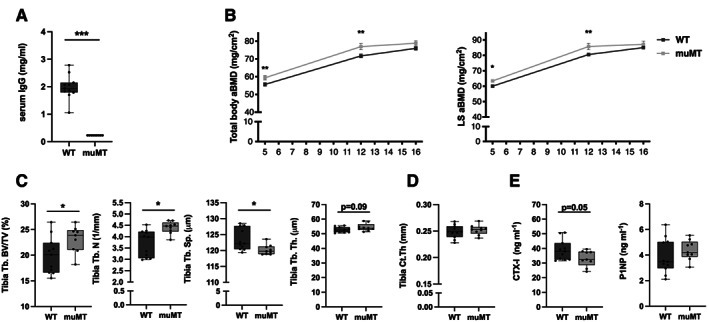
Male mice that lack mature B cells and immunoglobulins have increased trabecular bone. (*A*) IgG levels in serum. (*B*) Total body aBMD and LS L_3_–L_6_ aBMD were determined with DXA at 5‐week‐old, 12‐week‐old, and 16‐weeks of age in male muMT mice and WT littermates. *Indicates significant difference determined with Student's *t* test. (*C*) Trabecular BV/TV, Tb.N, Tb.Sp, and Tb.Th. (*D*) Ct.Th analyzed with high‐resolution μCT at 16 weeks of age. (*E*) Serum levels of P1NP (bone formation marker) and CTX (bone resorption marker). Student's *t* test was used to assess the differences between WT and muMT mice. *n* = 8–11. *p < 0.05, **p < 0.01, ***p < 0.001. aBMD = areal BMD; BV/TV = bone volume per total volume; Ct.Th = cortical thickness; LS = lumbar spine; Tb.N = trabecular number; Tb.Sp = trabecular separation; Tb.Th = trabecular thickness; WT = wild‐type.

To demonstrate that immunoglobulin deficiency is involved in the increased trabecular bone density in muMT mice, we performed an experiment where muMT mice were reconstituted with heat‐activated polyclonal IgGs locally in the knee joint. Periarticular bone in the proximal metaphysis of the tibia was analyzed using μCT. Trabecular BV/TV was significantly decreased after intraarticular injection with activated IgGs compared to injection with PBS (Fig. S4*A*), demonstrating that activated IgGs can counteract the increase in trabecular bone in muMT mice. Cortical thickness in the diaphyseal part of the tibia was not altered after intraarticular injection of heat‐activated IgG in muMT mice (Fig. S4 *B*).

**Fig. 4:**
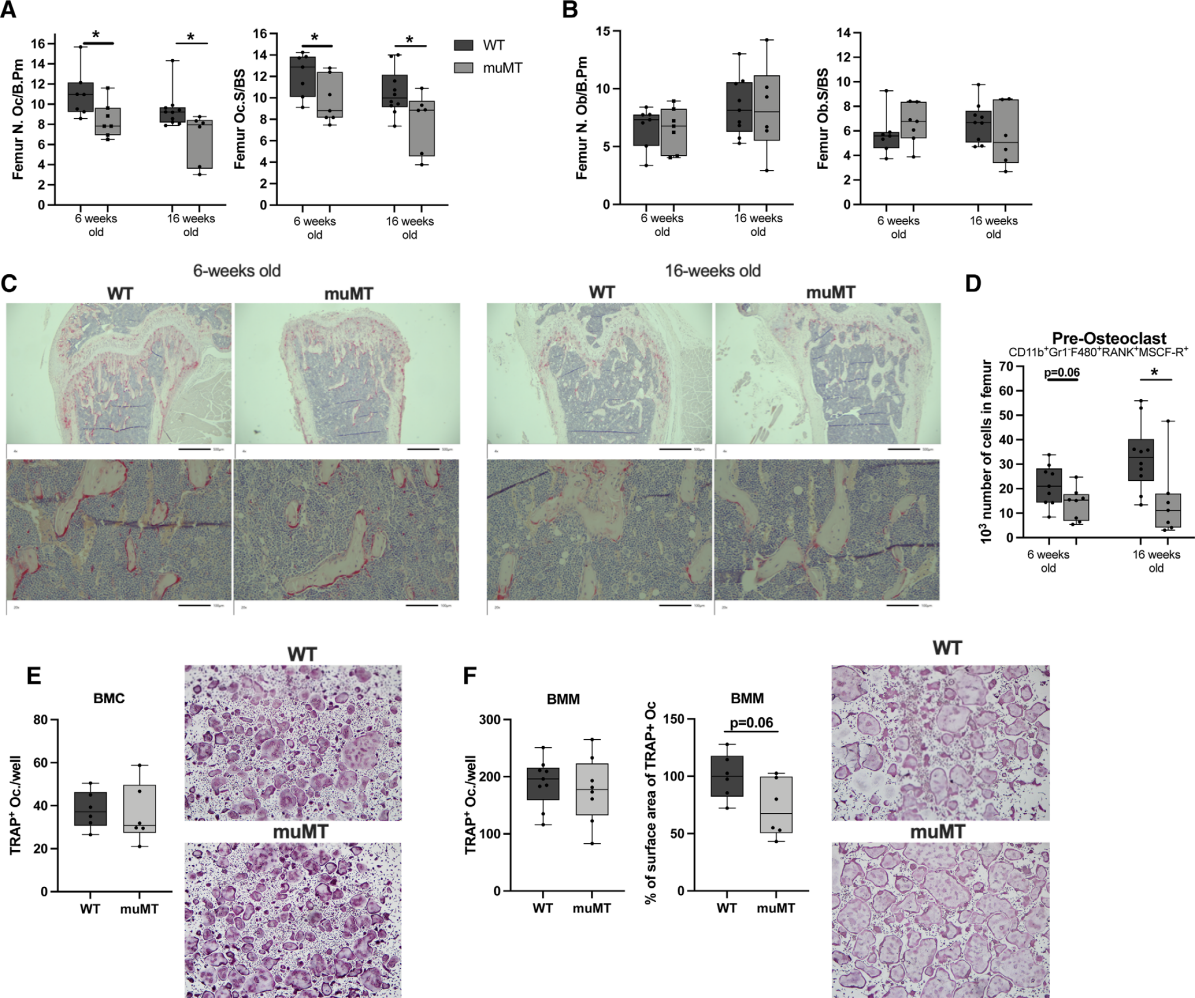
Reduced number of osteoclasts in mice lacking mature B cells and immunoglobulins. (*A*) The N.Oc/B.Pm and Oc.S/BS in the distal metaphyseal part of femur of 6‐week‐old and 16‐week‐old female muMT and WT littermate mice. Student's *t* test was used to assess differences between WT and muMT mice. *n* = 6–10. *p < 0.05. (*B*) The N.Ob/B.Pm and Ob.S/BS in the distal metaphyseal part of femur of 6‐week‐old and 16‐week‐old female muMT and WT littermate mice. Student's *t* test was used to assess the differences between WT and muMT mice. *n* = 6–10. (*C*) Representative images of TRAP‐positive osteoclasts in the femur. (*D*) The number of pre‐osteoclasts (CD11b+, Gr1−, F480+, RANK+, MCSF‐R+) in bone marrow of femur in 6‐week‐old and 16‐week‐old female muMT and WT mice. Student's *t* test was used to assess differences between WT and muMT mice at each time point. *n* = 8–10. *p < 0.05. *Ex vivo* RANKL stimulated osteoclast differentiation cultures from (*E*) crude BMC, *n* = 6, and (*F*) BMM, *n* = 8–9. Student's *t* test was used to assess the differences between WT and muMT mice. BMC = bone marrow cells; BMM = bone marrow macrophages; N.Ob/B.Pm = number of osteoblasts per bone perimeter; N.Oc/B.Pm = number of osteoclasts per bone perimeter; Ob.S/BS = osteoblast surface per bone surface; Oc.S/BS = osteoclast surface per bone surface; WT = wild‐type.

We also analyzed 16‐week‐old male muMT mice and found similar results as for the females. IgG levels in serum, determined with ELISA, were significantly reduced in male muMT mice compared to WT littermates (Fig. 3*A*). Total body and lumbar spine aBMD were increased in muMT mice compared to WT littermates at 5 and 12 weeks of age, while no significant differences were found at 16 weeks of age (Fig. 3*B*). Tibia trabecular BV/TV and trabecular number were increased in male muMT mice compared to WT and trabecular separation was decreased (Fig. 3*C*). The cortical thickness was not altered in the male mice (Fig. 3*D*). The serum marker of bone resorption, CTX‐I, showed a strong tendency to decrease in the muMT male mice, while no change in the bone formation marker P1NP was observed (Fig. 3*E*).

### Osteoclast number in trabecular bone is decreased in muMT mice

The increase in trabecular bone and reduction in serum CTX‐I indicate that the osteoclasts, rather than the osteoblasts, are affected in muMT mice. However, we investigated both the osteoclast and osteoblast surface and number *in vivo* in sections of femur metaphyseal trabecular bone in 6‐week‐old and 16‐week‐old female mice. Osteoclast surface and number were lower in the metaphyseal bone in muMT compared to littermates WT mice (Fig. 4*A*,*B*), whereas the osteoblast surface and number were unaltered (Fig. 4*B*,*C*). Furthermore, the number of osteoclast precursors was reduced in 16‐week‐old female muMT mice compared to WT littermates and there was a tendency toward a reduction at 6 weeks old in muMT mice as defined with flow cytometry (Fig. 4*D*). There were no differences in the numbers of osteoclasts after *ex vivo* stimulation with RANKL from total BMCs or from BMMs (Fig. 4*E*,*F*). However, a tendency toward reduced BMM osteoclast surface area was detected in muMT mice compared to WT littermates (Fig. 4*F*).

### Alterations in osteoclast‐related gene expression in vivo

To further understand the mechanism behind the bone phenotype, we investigated the messenger RNA (mRNA) expression in BM and vertebral bodies from 6‐week‐old and 16‐week‐old females, as well as from 16‐week‐old male muMT and WT mice. In the 6‐week‐old female mice, *OPG* expression was dramatically reduced in both BM and vertebral bodies from muMT mice compared to WT littermates (Table 1). *RANKL* expression was upregulated in muMT mice compared with WT mice in BM and a tendency was also seen in vertebral bodies (Table 1). The decrease in *OPG*, together with the increase in *RANKL* resulted in a dramatic increase in the *RANKL/OPG* ratio in both BM and vertebral bodies in muMT mice compared to WT littermates. In BM, a reduction in *IL‐17* expression was found in muMT mice compared to WT littermates, but there were no alterations in *IL‐6*, *TNFα*, *IL‐23a*, *RorC*, *CSFR*, *NFATc1*, or *CX3CR1* expression. In vertebral bodies, the expression of the osteoclast‐related genes *NFATc1* and *TRAP* were downregulated in muMT mice compared to WT littermates, whereas the expression of *ALP*, an osteoblast‐related gene, was unaltered. The same patterns as described above were also displayed in 16‐week‐old female mice as well as in 16‐week‐old male mice (Table S2). In addition, gene expression analyses of several osteoblast‐related genes (*osteocalcin*, *Col2alpha1*, *Runx2*, *ALP*) in *ex vivo* cultured osteoblasts revealed that there were no differences between muMT and WT mice (Table S3).

**Table 1:** Expression Pattern in BM and Vertebral Bodies

Variable	WT	muMT
BM		
OPG	1.00 ± 0.66	0.44 ± 0.17*
RANKL	1.00 ± 0.40	2.29 ± 0.29*
RANKL/OPG	1.00 ± 0.41	14.13 ± 6.21*
CSFR	1.00 ± 0.17	1.26 ± 0.14
IL‐6	1.00 ± 0.32	0.89 ± 0.03
IL‐17	1.00 ± 0.03	0.79 ± 0.06*
IL‐23	1.00 ± 0.05	1.04 ± 0.08
TNFα	1.00 ± 0.29	0.81 ± 0.11
RorC	1.00 ± 0.17	1.33 ± 0.14
NFATc1	1.00 ± 0.10	1.04 ± 0.16
Cx3Cr1	1.00 ± 0.12	0.96 ± 0.14
Vertebral bodies		
OPG	1.00 ± 0.08	0.67 ± 0.07*
RANKL	1.00 ± 0.11	1.33 ± 0.10#
RANKL/OPG	1.00 ± 0.10	1.70 ± 0.19*
RANK	1.00 ± 0.06	0.93 ± 0.07
NFATc1	1.00 ± 0.22	0.40 ± 0.09*
TRAP	1.00 ± 0.10	0.75 ± 0.05*
ALP	1.00 ± 0.18	1.08 ± 0.08

RNA expression in BM and vertebral bodies in 6‐week‐old female WT and muMT mice. WT mice (control) are set to 1, and expression in muMT mice is related to the WT littermate controls and displayed as fold change. *n* = 8–9.

ALP = alkaline phosphatase; BM = bone marrow; CSFR = colony stimulating factor receptor; Cxcr1 = chemokine C‐X2‐C motif receptor 1; IL = interleukin; NFATc1 = nuclear factor of activating T cells 1; OPG = osteoprotegerin; RANK = receptor activator of nuclear factor kappa‐B; RANKL = RANK‐ligand; RoRC = RAR‐related orphan receptor C; NFκB = nuclear factor kappa‐B; TNFα = tumor necrosis factor alpha; TRAP = tartrate resistant acid phosphatase; WT = wild type.

# = 0.07, *p < 0.05, Student's *t* test.

### Estrogen deficiency reduces bone mass in mice lacking mature B cells and immunoglobulins

**Fig. 5:**
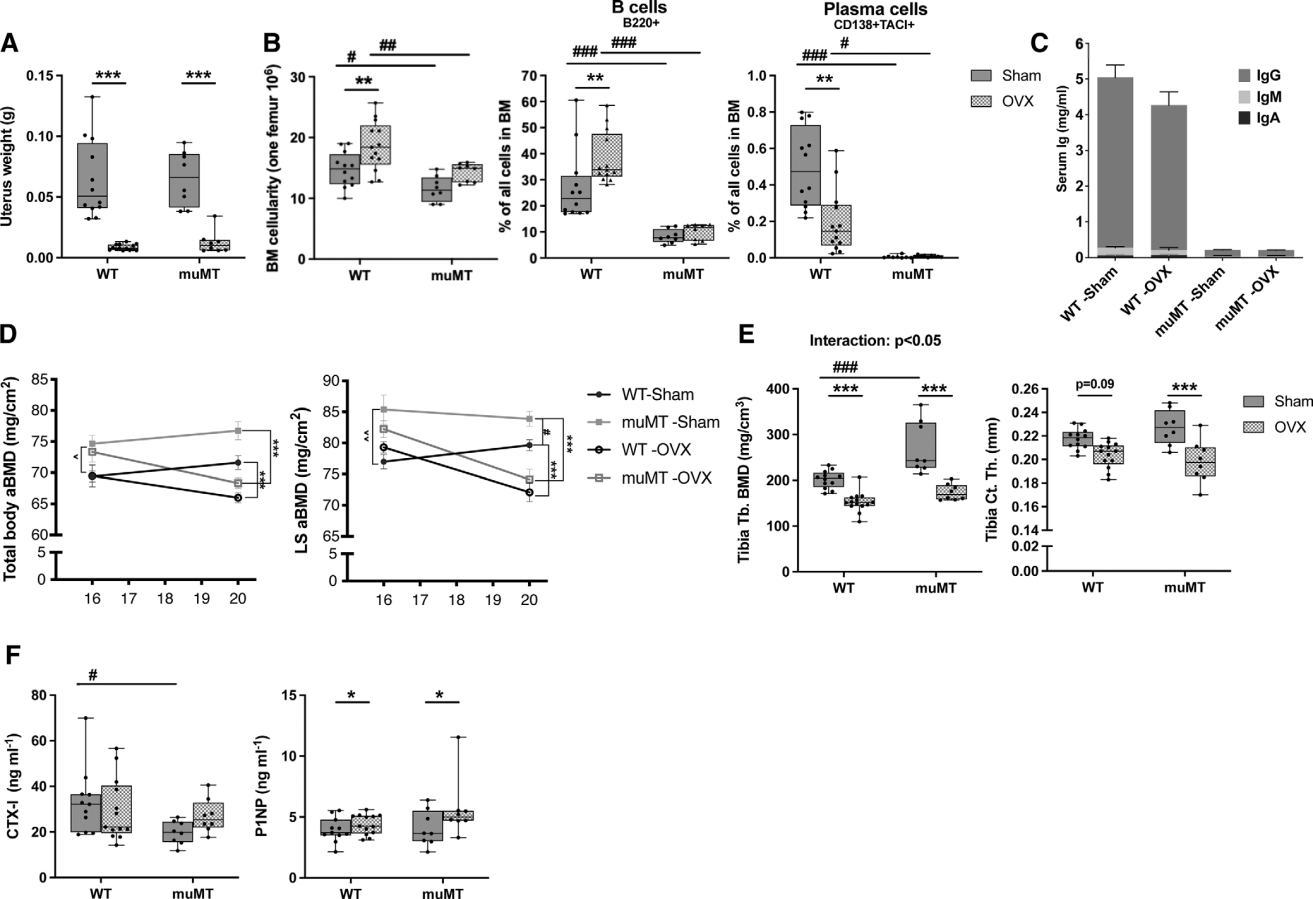
The lack of B cells and immunoglobulins does not prevent bone loss caused by estrogen deficiency. Female muMT and WT littermate mice were ovx or sham‐operated at 16 weeks of age and euthanized after 4 weeks. (*A*) Uterus weight and (*B*) BM cellularity and B cell and plasma cell frequencies in BM. (*C*) Serum immunoglobulin levels are presented as interlined boxes, where IgG, IgA, and IgM levels are displayed together. (*D*) Total body aBMD, LS L_3_–L_6_ aBMD before and 4 weeks after the operation. (*E*) Tb BMD and Ct.Th in tibia assessed by pQCT. (*F*) Serum levels of CTX (bone resorption marker) and P1NP (bone formation marker). *n* = 8–14. Student's *t* test was used to assess the differences between all WT and all muMT mice before operation. ^*p* < 0.05, ^^*p* < 0.01. At termination statistical analyses were performed using ANOVA and Tukey's post hoc test (B cells, LS aBMD, Tb.BMD, Ct.Th, P1NP, and CTX), ANOVA and Dunnett's T3 post hoc test for data with unequal variance (uterus weight and plasma cells) or using ANCOVA and Tukey's post hoc when there was an interexperiment variation (BM cellularity and total aBMD). WT versus muMT mice, #*p* < 0.05, ##*p* < 0.01, ###*p* < 0.001, and ovx versus sham, *p < 0.05, **p < 0.01, ***p < 0.001. The interaction between the genotype (muMT and WT) and intervention (sham and ovx‐operation) was calculated using a mixed‐model two‐way ANOVA. aBMD = areal bone mineral density; BM = bone marrow; BMD = bone mineral density; Ct.Th = cortical thickness; LS = lumbar spine; ovx = ovariectomized; Tb = trabecular; WT = wild‐type.

muMT and WT littermates were ovariectomized (ovx) to mimic a postmenopausal status, or sham‐operated at 16 weeks of age. Four weeks after ovx the depletion of endogenous estrogen levels was verified with decreased uterus weight in both muMT and WT mice (Fig. 5*A*). BM cellularity and B cell (B220^+^) frequency were increased, whereas the frequency of plasma cells decreased after ovx compared to sham‐operation in WT mice (Fig. 5*B*). Serum immunoglobulin levels were not significantly affected by ovx (Fig. 5*C*). BM cellularity, B cells, plasma cells, as well as immunoglobulins were lower in muMT mice compared to WT littermates both in sham‐operated and ovx mice at 20 weeks of age (Fig. 5*B*,*C*).

Four weeks after the ovx procedure, total body and lumbar spine aBMD were reduced in both WT and muMT mice to a similar extent compared to sham animals (Fig. 5*D*). Using pQCT analysis we confirmed that ovx reduced both trabecular BMD and cortical thickness in muMT mice (Fig. 5*E*). In WT mice, ovx significantly reduced trabecular BMD and there was a tendency toward a decrease cortical thickness. Similar to what is seen in Fig. 2*A*, lumbar spine aBMD in muMT mice was higher compared to WT mice at both 16 and 20 weeks of age in sham‐operated animals (Fig. 5*D*). Trabecular BMD was also higher in sham‐operated muMT mice compared to sham‐operated WT littermates at 20 weeks of age, but there was no difference in trabecular BMD between ovx muMT and ovx WT mice (Fig. 4*E*). The bone loss caused by ovx was similar between WT and muMT mice (total body aBMD and lumbar spine aBMD), or slightly higher (trabecular BMD, *p* < 0.05) as determined by two‐way ANOVA (interaction factor). In addition, the lower CTX‐1 levels seen in muMT mice compared to WT mice in 6‐week‐old and 16‐week‐old‐mice (Fig. 2*D*) as well as in sham‐operated mice at 20 weeks of age were not seen in ovx mice (Fig. 5*F*). P1NP serum levels were affected by ovx but not the lack of mature B cells and immunoglobulins (Fig. 5*F*).

### B cell and immunoglobulin deficiency do not affect arthritis development or immune‐mediated bone loss

**Fig. 6:**
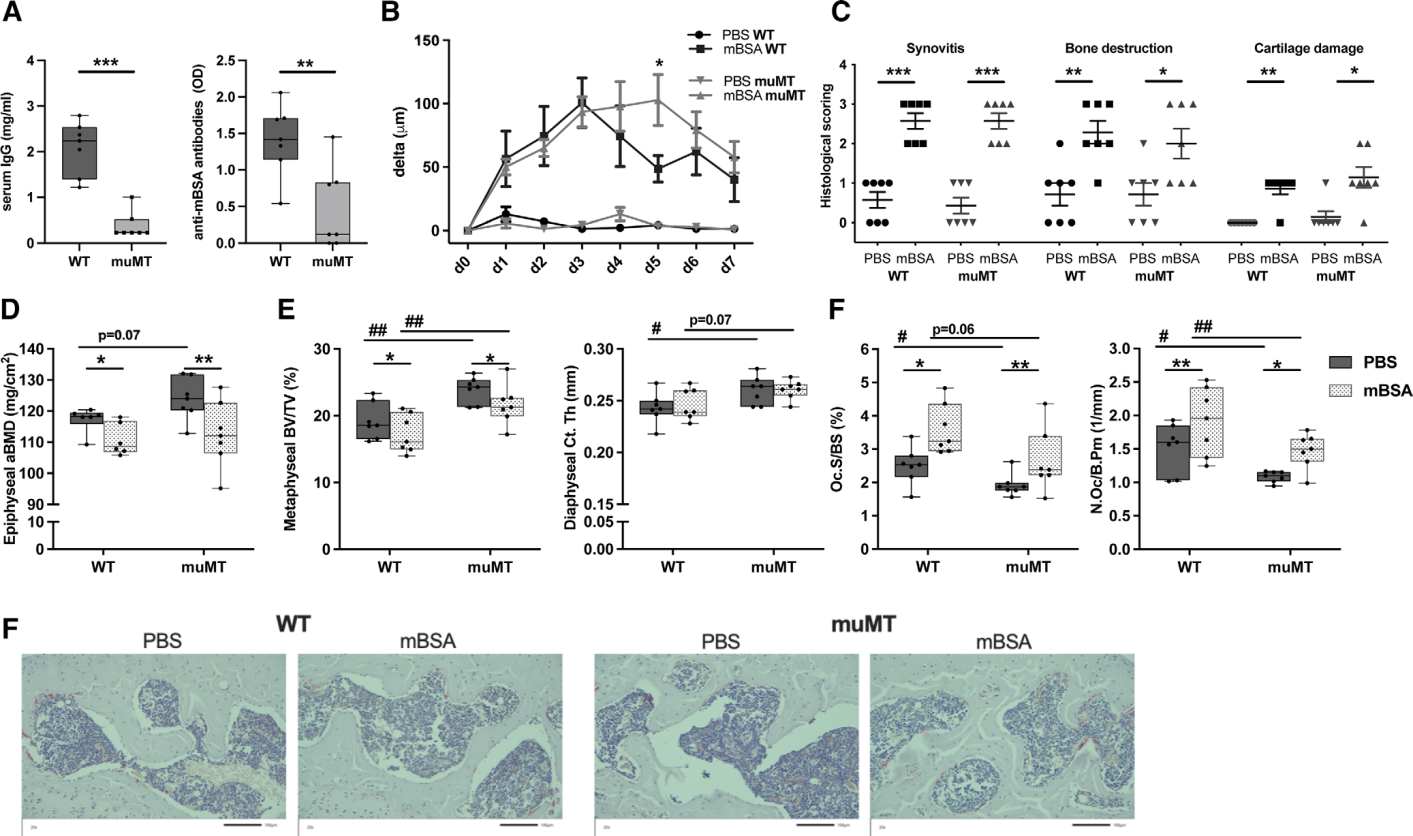
Mature B cells and immunoglobulins are dispensable for arthritis‐induced bone loss. Male muMT and WT littermate mice were immunized with mBSA. Antigen challenge was repeated intraarticularly after 7 days and knee joint swelling was measured daily over 7 days. (*A*) Serum IgG and anti‐mBSA antibodies were investigated at termination. Statistical analysis was performed using Student's *t*‐test, **p < 0.01, ***p < 0.001, *n* = 7. (*B*) Difference in swelling over the knee from baseline in micrometers (μm) in muMT and WT mice challenged with mBSA or PBS. Statistical analysis was performed at each measurement time point using Student's *t* test, *p < 0.05, *n* = 7. (*C*) Histological scoring (0–3) of synovitis, bone destruction, and articular cartilage damage 14 days after immunization. Statistical analysis was performed with Mann‐Whitney test in each genotype, *p < 0.05, **p < 0.01, ***p < 0.001, *n* = 7. (*D*) Epiphyseal aBMD was defined with DXA in the tibia. *n* = 6–7. (*E*) Diaphyseal Ct.Th and trabecular metaphyseal BV/TV in tibia were defined with high‐resolution μCT. (*F*) N.Oc/B.Pm and Oc.S/BS in the epiphyseal part of the tibia. *n* = 7. At termination Student's *t* test was performed between genotypes, WT and muMT, #*p* < 0.05, ##*p* < 0.01, and paired *t* test between intervention in the same mouse (non‐arthritic versus arthritic side), *p < 0.05, **p < 0.01. The interaction between the genotype (muMT and WT) and intervention (non‐arthritic and arthritic side) was calculated using a mixed‐model two‐way ANOVA. aBMD = areal bone mineral density; BV/TV = bone volume per total volume; Ct.Th = cortical thickness; mBSA = methylated bovine serum albumin; N.Oc/B.Pm = number of osteoclasts per bone perimeter; Oc.S/BS = osteoclast surface per bone surface; WT = wild‐type.

It has previously been reported that B cells and immunoglobulins are dispensable for the development of AIA.^(^ ^30^ ^)^ The muMT mice displayed severely decreased serum levels of IgG as well as decreased levels of serum mBSA specific antibodies compared to WT mice after mBSA induction in AIA mice (Fig. 6*A*). The swelling over the arthritic knee was increased in muMT mice compared to WT mice 5 days after the mBSA intraarticular injection, but no difference was seen when analyzing total area under the curve, area under the initial part of arthritis development (day 0–day 3), or when the arthritis was established (day 4–day 7) (Fig. 6*B*). Similarly, no differences in histological scoring of arthritis were detected between muMT and WT mice (Fig. 6*C*).

Induction of arthritis resulted in local arthritic bone loss, displayed as reduced trabecular epiphyseal aBMD in the tibia, to a similar extent in muMT mice and WT littermates (Fig. 6*D*). The arthritis‐induced bone loss was confirmed using μCT of the trabecular metaphyseal BV/TV (Fig. 6*E*). The cortical bone in the tibia was not affected by the local arthritis induction in either WT or muMT mice, but cortical thickness was increased in the tibia of the nonarthritic muMT leg compared to the nonarthritic WT leg, and a tendency towards an increase was also seen in muMT mice compared to WT mice when comparing the tibia of the arthritic legs (Fig. 6*E*). Arthritis induction resulted in stimulation of osteoclasts in both WT and muMT mice, as shown by increased osteoclast number and surface in the epiphyseal part of the tibia in the arthritic knee compared to the nonarthritic knee (Fig. 6*F*). muMT mice showed a reduced number of osteoclasts as well as decreased osteoclast surface compared to WT mice in the tibia epiphysis of the PBS‐injected (nonarthritic) knee (Fig. 6*F*), and similar results were seen when analyzing osteoclast parameters in the metaphyseal part of the PBS‐injected (nonarthritic) knees (Fig. S5). The number of osteoclasts was also significantly decreased in muMT mice compared to WT mice in the arthritic knee in the epiphysis, and a tendency towards a decrease was also seen for osteoclast surface (Fig. 6*F*).

## Discussion

The present study investigates the role of mature B cells and immunoglobulin secretion in the regulation of bone in healthy and pathogenic conditions. We show that the absence of mature B cells and immunoglobulins leads to increased trabecular bone mass and this effect is associated with inhibition of osteoclasts.

Previous animal models of B cell deficiency have displayed conflicting results regarding effects on bone.^(^ ^18^, ^19^, ^20^ ^)^ Li and colleagues^(^ ^19^ ^)^ reported reduced trabecular and cortical parameters in the muMT model and explained that these effects are associated with the decrease in B cell production of OPG, the RANKL decoy receptor. In accordance with Li and colleagues,^(^ ^19^ ^)^ we also found a reduction of *OPG* mRNA in BM and vertebral bodies as well as a higher *RANKL/OPG* ratio. However, in contrast, we found an increased trabecular bone density. We also found fewer osteoclasts and decreased serum levels of the bone resorption marker CTX, as well as a reduction in expression of several osteoclast‐related genes in the muMT mice compared to WT littermates. These conflicting data indicate a complex relationship between mature B cells, immunoglobulins, and regulation of the skeleton. RANKL and OPG are important factors regulating the maturation and activation of osteoclasts, but in our settings, other factors had a stronger impact on bone remodeling because the trabecular bone was increased despite a significant increase in the *RANKL/OPG* ratio. Osteoclasts express FcγRs,^(^ ^5^, ^12^ ^)^ and several studies from us and others have shown that activated IgG, which can bind FcγRs, has the capacity to influence osteoclast development.^(^ ^4^, ^5^, ^10^, ^11^, ^12^, ^14^, ^15^ ^)^ The muMT mice have severely reduced levels of immunoglobulins and thereby a diminished stimulation of FcγRs. This might, at least partially, explain the increase in trabecular bone and decrease in number of osteoclasts seen in the muMT mice.

The main difference between this study and previous studies in muMT mice is the use of inbred WT littermate controls. Small genetic variations, and variations in the housing conditions during breeding, may influence the skeletal tissue and the use of WT littermate controls limits this variation. We verified the lack of mature B cells and plasma cells in the inbred muMT mice and confirmed that the inbred muMT mice lack immunoglobulin‐producing cells and have reduced levels of serum IgG, IgA, and IgM. As Macpherson and colleagues^(^ ^37^ ^)^ have described, the IgA serum levels in muMT were significantly reduced compared to WT mice, but not completely absent, which is the case for IgG and IgM levels. Furthermore, we found normal levels of both pro‐B and pre‐BI cells, which are early‐stage B cells still not expressing IgM, in bone marrow, whereas mature B cells, including plasma cells, were reduced.

We conducted a thorough investigation of bone parameters and found a consistent increase in trabecular bone density in prepubertal (6‐week‐old), pubertal (12‐week‐old), as well as in young adult (16‐week‐old) female muMT mice compared to WT littermates in both the appendicular (tibia) and the axial (vertebrae) skeleton. This increase in trabecular density was also seen in 16‐week‐old male mice, and in the AIA experiment. Local reconstitution of activated IgGs in muMT mice resulted in decreased trabecular bone density, demonstrating that IgG deficiency most likely is involved in the trabecular bone phenotype in muMT mice. Cortical bone was unaltered between muMT and WT mice in the 6‐week‐old, 12‐week‐old, and 16‐week‐old female mice, the 16‐week‐old male mice, and the mice in the ovx experiment, whereas an increase in cortical thickness was detected in muMT mice compared to WT mice in the AIA experiment. Thus, taken together, reduction of mature B cells and immunoglobulins seems to have a greater impact on trabecular bone than cortical bone. Cortical and trabecular bone share the same cell types but have considerable structural differences. This may explain the different effects of the lack of mature B cells and immunoglobulins between the two bone compartments. Our finding contrasts with the decrease in both cortical and trabecular bone mass, which was previously reported by Li and colleagues^(^ ^19^ ^)^ and Khass and colleagues,^(^ ^20^ ^)^ and this discrepancy might be dependent on the use of different types of controls (inbred versus separately bred). Furthermore, Khass and colleagues^(^ ^20^ ^)^ reported a reduced number of osteoblasts in the trabecular bone, but in our settings, no alteration in osteoblast number in the bone sections or in cell cultures between muMT and WT mice were detected.

In the absence of mature B cells, the muMT mice displayed higher frequencies of other immune cells, such as T cells, neutrophils, and monocytes, but due to decreased cellularity, there were no changes observed in the absolute number. However, the alteration in frequencies of immune cells might influence the inflammatory status and therefore we investigated pro‐inflammatory cytokines in BM, which may affect osteoclasts. We found that the *TNFα*, *IL‐23*, and *IL‐6* expression levels were unchanged. However, there was a significant reduction in *IL‐17* expression, and because IL‐17 is a known activator of osteoclastogenesis,^(^ ^38^ ^)^ this may be involved in the reduced number of osteoclasts seen in muMT mice. A limitation of this study is that it is not determined which cell type is responsible for the decreased expression of *IL‐17*. Markers for Th17 cells were not included in the FACS analysis; however, the transcription levels of RorC, which is expressed by Th17 cells, and IL23, which is a cytokine‐promoting Th17 proliferation and IL17 production, were not altered between the WT and muMT mice. These data indicate that the change in *IL17* expression is not due to altered IL17 expression in Th17 cells, but to altered expression in some other IL17‐expressing cell type. Further studies are needed to determine the source of the altered *IL17* expression in the bone marrow of muMT mice.

In addition to the decrease in the number of osteoclasts present in muMT mice compared to littermate controls, there were decreased frequencies and absolute numbers of pre‐osteoclasts in the BM of muMT mice. A tendency to decreased osteoclast surface area was also detected in BMM‐derived osteoclasts from muMT mice compared to osteoclasts from WT littermates. However, no difference in osteoclast count between muMT and WT littermates after RANKL stimulation of BM cells *ex vivo*. One possible explanation for this discrepancy is that the *ex vivo* experimental design is not sensitive enough to detect differences in pre‐osteoclast numbers. However, because we and others previously have shown that activated IgG can enhance RANKL‐mediated osteoclastogenesis both in cultivation and *in vivo*,^(^ ^4^, ^5^, ^10^, ^11^, ^12^ ^)^ another explanation might be that the effect on osteoclast numbers *in vivo* is more dependent on factors secreted from B cells, such as immunoglobulins, than on differences in pre‐osteoclast number. Furthermore, the number of osteoclasts formed after *ex vivo* RANKL stimulation of a fixed number of BM macrophages was similar between WT and muMT mice, indicating that the muMT mutation has no cell autonomous effect on pre‐osteoclast differentiation capability. We also investigated *Cx3cr1* expression, which is an indicator of the presence of inflammation‐induced osteoclasts,^(^ ^39^ ^)^ in BM. *Cx3cr1* expression levels were unchanged between muMT and WT mice, which indicates that inflammation‐induced osteoclasts are not affected by the loss of immunoglobulins and mature B cells.

The lack of immunoglobulins and mature B cells resulted in increased trabecular bone density and fewer osteoclasts in healthy conditions, and we also wanted to determine the importance of mature B cells and immunoglobulins in states of induced bone loss. Bone loss is common after menopause, so we wanted to determine the role of mature B cells and immunoglobulins in bone loss seen after estrogen deficiency using the muMT model with WT littermate controls. Estrogen deficiency is known to result in an increase in B cell frequency and reduced plasma cell frequency, and this was confirmed after ovx in our WT mice. Using separately bred WT controls, Li and colleagues^(^ ^23^ ^)^ previously showed that lack of mature B cells and immunoglobulins have no impact on ovx‐induced bone loss, We also found that ovx was able to significantly decrease bone mass in muMT mice, supporting the finding by Li and colleagues.^(^ ^23^ ^)^ Sham‐operated muMT mice had increased bone mass and decreased serum markers of bone resorption at 20 weeks of age compared to WT littermates. Serum levels of the bone resorption marker CTX‐I were not affected 4 weeks after ovx in either WT or muMT mice, whereas levels of the bone formation marker P1NP were significantly elevated. Ovx is known to induce an early increase in both bone resorption and bone formation markers in serum, indicating a high bone turnover^(^ ^40^ ^)^ and our data suggests that in our settings, a new steady state is reached for bone resorption 4 weeks after ovx. Interestingly, these differences between muMT and WT littermates were abolished in ovx mice, indicating that estrogen deprivation has a superior effect compared to mature B cells and immunoglobulin deficiency on the skeletal tissue.

Immune activation and arthritis are associated with bone loss. Formation of autoantibodies and IgG‐complex deposition in articular tissues elicit a local immune response that plays a crucial role in the pathogenesis of RA and leads to local bone destruction.^(^ ^14^, ^41^, ^42^ ^)^ Furthermore, mice lacking activating FcγRs have inhibited osteoclast activation^(^ ^5^, ^11^ ^)^ as well as reduced bone destruction after arthritis induction using the KBxN serum‐induced arthritis model,^(^ ^5^ ^)^ suggesting that immunoglobulin activation can affect immune‐induced bone loss. B cells are recognized in the articular tissue and anti‐B cell treatment reduces the inflammation status, however, conflicting results are reported for the skeleton.^(^ ^43^, ^44^ ^)^ To further investigate the importance of B cells and immunoglobulins for immune‐mediated bone loss, we induced arthritis in muMT mice and WT littermate controls. We used the AIA model, a mainly T cell‐driven monoarthritis model,^(^ ^30^ ^)^ which is shown to result in both bone erosions and local immune‐mediated periarticular bone loss.^(^ ^32^ ^)^ The muMT mice with AIA, which have severely diminished immunoglobulin levels and reduced mBSA‐specific antibodies, developed the same degree of swelling and histological signs of arthritis, including bone erosions, compared to WT littermates. These data support previous reports demonstrating that the development of arthritis and bone erosions, using the AIA model, is not affected by the absence of mature B cells and immunoglobulins.^(^ ^30^, ^45^ ^)^ Furthermore, a similar reduction of trabecular bone and increased number of osteoclasts in the periarticular area after arthritis induction was detected in muMT mice and WT littermates, demonstrating that immune‐mediated periarticular bone loss in the AIA model does not require mature B cells and immunoglobulins. Interestingly, the trabecular bone in the metaphyseal area of the tibia, as well as osteoclast number, were higher in muMT mice compared to WT littermates also after arthritis induction. These data indicate that despite the increased inflammatory state in the arthritic knee, the lack of immunoglobulins and mature B cells can still affect the trabecular bone mass and the number of osteoclasts. This contrasts with the bone loss caused by estrogen deficiency, which resulted in a trabecular bone density in muMT mice that was not significantly different from the bone density in WT littermates.

In summary, our data show that mice deficient in mature B cells and immunoglobulins have increased trabecular bone density and a reduction of osteoclasts, suggesting an important role for mature B cells and immunoglobulins in the normal physiological regulation of trabecular bone, whereas they seem to be dispensable for estrogen‐deficiency and arthritis‐mediated bone loss.

## Author Contributions

**Marie Kristina Lagerquist:** Conceptualization; data curation; formal analysis; funding acquisition; investigation; methodology; project administration; supervision; validation; visualization; writing – original draft; writing – review and editing. **Priti Gupta:** Data curation; formal analysis; methodology; writing – original draft; writing – review and editing. **Edina Sehic:** Data curation; methodology; writing – review and editing. **Karin L. Horkeby:** Data curation; methodology; visualization; writing – review and editing. **Julia M. Scheffler:** Data curation; writing – review and editing. **Jauquline Nordqvist:** Data curation; writing – review and editing. **Lina Lawenius:** Data curation; writing – review and editing. **Ulrika Islander:** Data curation; supervision; writing – review and editing. **Carmen Corciulo:** Data curation; methodology; writing – review and editing. **Petra Henning:** Data curation; formal analysis; investigation; methodology; writing – original draft; writing – review and editing. **Hans Carlsten:** Conceptualization; funding acquisition; project administration; resources; supervision; writing – review and editing. **Cecilia Engdahl:** Conceptualization; data curation; formal analysis; funding acquisition; investigation; methodology; project administration; supervision; validation; visualization; writing – original draft; writing – review and editing.

## Conflict of Interests

The authors declare that they have no competing interests.

### Peer Review

The peer review history for this article is available at https://publons.com/publon/10.1002/jbm4.10670.

## Supporting information


**Fig. S1** Gating strategies for bone marrow B cells. Singlets were determined using FSC‐Height versus FSC‐Area followed by live cell gating based on size and granularity. CD3− cells were gated into B cells (B220+), pro‐B cells (B220low Ckit+ CD19−), pre‐BI cells (B220low Ckit+ CD19+), immature‐B cells (B220low IgM+) and plasma cells (CD138+ TACI+). The same gating strategy was used in spleen for B cells and plasma cells.

**Fig. S2** Gating strategies for bone marrow pre‐osteoclasts. Singlets were determined using FSC‐Height versus FSC‐Area followed by live cell gating based on size and granularity. CD3‐ cells were gated into CD11b + followed by Gr1‐F480 +, followed by MSCF‐R + and RANK +.

**Fig. S3** Female mice that lack mature B cells and immunoglobulins have increased trabecular bone in the axial skeleton. Trabecular bone volume per total volume (BV/TV) was analyzed with high‐resolution micro‐CT in vertebrae L5 in 6‐ and 16‐week old female mice. Student's *t*‐test was used to assess the differences between WT and muMT mice. *N* = 7–10. *p < 0.05.

**Fig. S4** Polyclonal heat‐activated IgGs rescue the trabecular bone phenotype in muMT mice. (a) MuMT mice were intraarticularly injected in one hind leg with polyclonal heat‐activated IgG, while the contralateral leg was injected with PBS. WT mice with an intraarticular injection with PBS were used for comparison. Tibia trabecular bone volume per total volume (BV/TV) in the metaphysis and (b) cortical thickness (Ct.Th) in the diaphysis were analyzed with high‐resolution micro‐CT. *N* = 7–10. *p < 0.05 versus muMT + PBS, Student's *t*‐test was used to assess the differences.

**Fig. S5** Osteoclast number and surface in metaphyseal bone in males. Male muMT and wild‐type (WT) littermate mice were immunized with methylated bovine serum albumin (mBSA). Antigen challenge was repeated intra‐articularly after 7 days. Number of osteoclasts per bone perimeter (N.Oc/B.Pm) and osteoclast surface per bone surface (Oc.S/BS) in the metaphyseal part of the tibia. *N* = 5–6. Unpaired Student's *t*‐test was used between WT and muMT, *#p* < 0.05, and paired *t*‐test was used for comparisons within the same mouse (non‐arthritic versus arthritic side), *p < 0.05.

**Table S1** Physiological Assessment of muMT Mice. Weights of organs in wild‐type (WT) and muMT mice. *N* = 7–12. Student's *t*‐test, WT versus muMT. g; gram, mg; milligram
**Table S2** Expression Pattern in Bone Marrow (BM) and Vertebral Bodies. RNA expression in BM and vertebral bodies in 16‐weeks old female and male wild‐type (WT) and muMT mice. WT mice (control) is set to 1, and expression in muMT mice is related to the WT littermates' controls and displayed as fold change. *N* = 9–12. # < 0.07, *p < 0.05, Student's *t*‐test. OPG, osteoprotegerin; RANK, receptor activator of nuclear factor kappa‐B; RANKL, RANK‐ligand; CSFR, colony stimulating factor receptor; IL, interleukin; TNFα, tumor necrosis factor alpha; RoRC, RAR‐related orphan receptor C; NFATc1, nuclear factor of activating T cells 1; Cxcr1, chemokine C‐X2‐C motif receptor 1; NFκB, nuclear factor kappa‐B; TRAP, tartrate resistant acid phosphatase; ALP, alkaline phosphatase.
**Table S3** Expression Pattern of Osteoblast‐Associated Genes. Ex vivo stimulated osteoblasts from the appendicular skeleton from wild‐type (WT) and muMT mice. WT mice (control) are set to 1, and the expression in muMT mice is related to the control group and displayed as fold change. *N* = 4–5. Col1α1, collagen type 1 alpha 1; Runx2, runt‐related transcription factor 2; ALP, alkaline phosphatase.

## Data Availability

The data that supports the findings of this study are available from the corresponding author upon request.
