# Supplementary figures and images for: Reduction of Mature B Cells and Immunoglobulins Results in Increased Trabecular Bone
Source: JBMR Plus. 2022 Aug 30;6(9):e10670. doi: 10.1002/jbm4.10670 (PMC9465004; doi:10.1002/jbm4.10670)

# Supplementary Figure 1

## Wild type

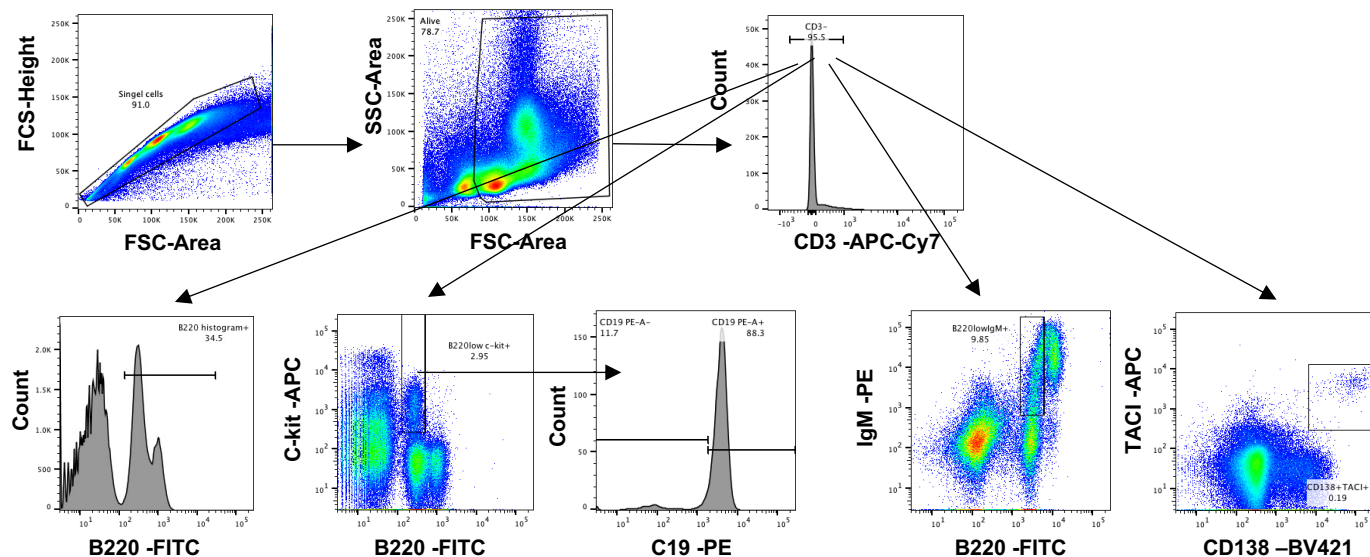

## muMT

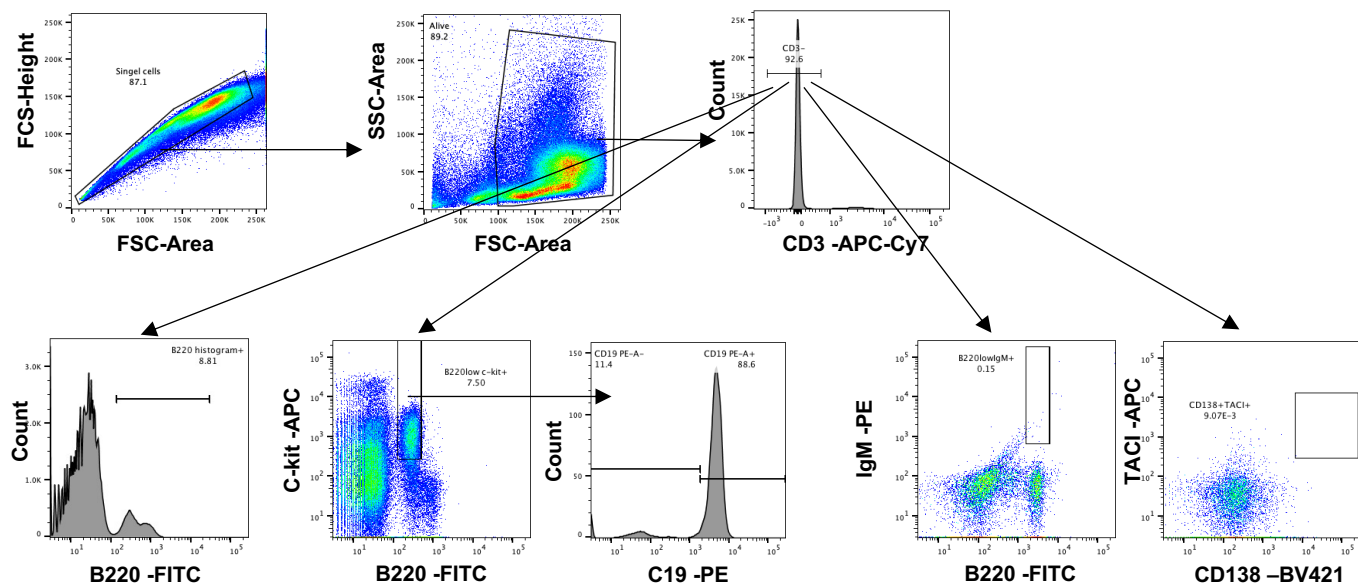

Supplement: Supplementary file 1 — Fig. S1 Gating strategies for bone marrow B cells. Singlets were determined using FSC‐Height versus FSC‐Area followed by live cell gating based on size and granularity. CD3− cells were gated into B cells (B220+), pro‐B cells (B220low Ckit+ CD19−), pre‐BI cells (B220low Ckit+ CD19+), immature‐B cells (B220low IgM+) and plasma cells (CD138+ TACI+). The same gating strategy was used in spleen for B cells and plasma cells. [file JBM4-6-e10670-s004.pdf]

# Supplementary Figure 2

Wild type

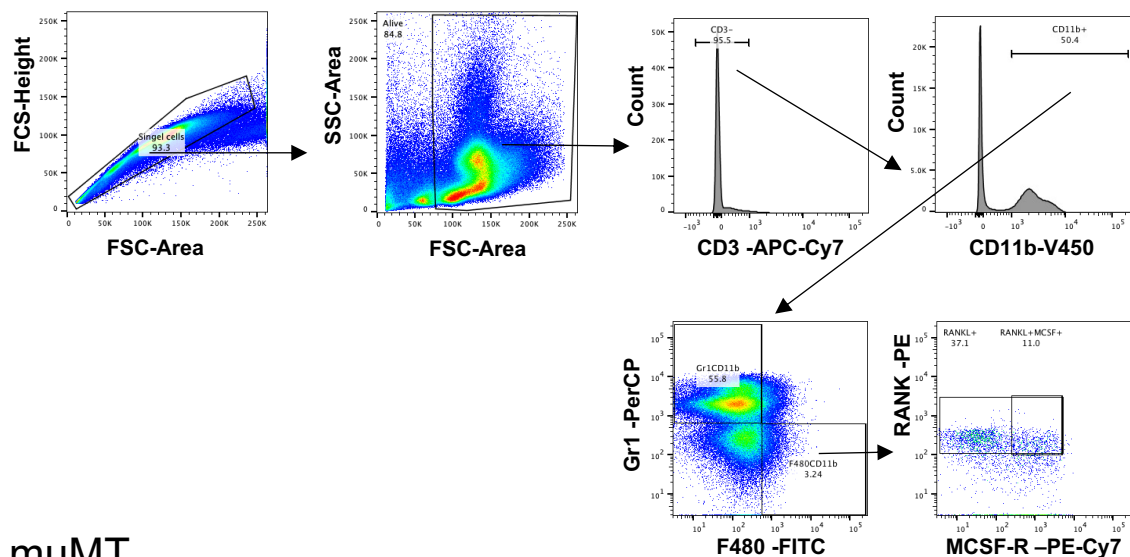

muMT

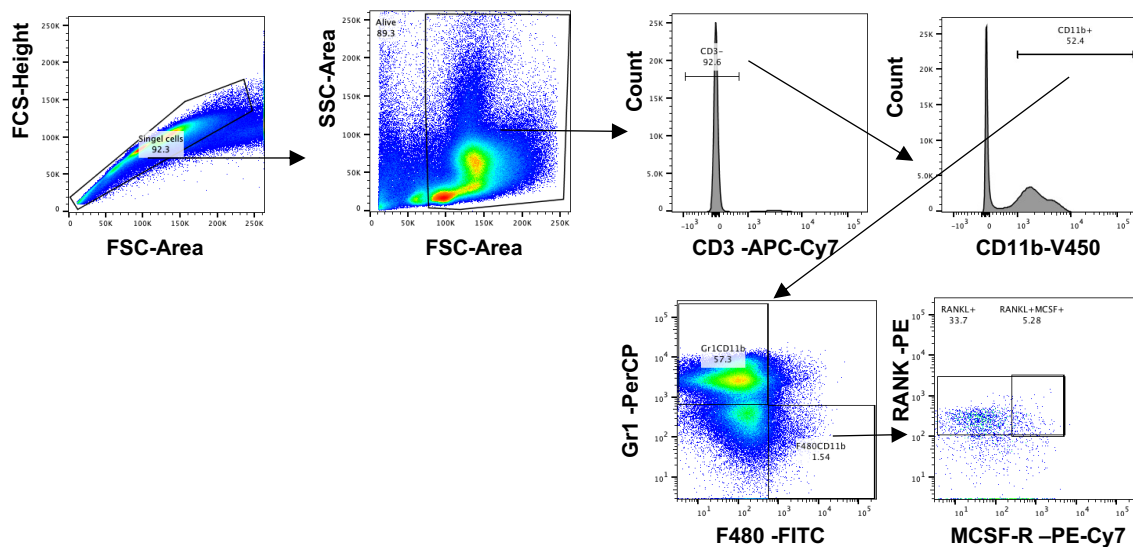

Supplement: Supplementary file 2 — Fig. S2 Gating strategies for bone marrow pre‐osteoclasts. Singlets were determined using FSC‐Height versus FSC‐Area followed by live cell gating based on size and granularity. CD3‐ cells were gated into CD11b + followed by Gr1‐F480 +, followed by MSCF‐R + and RANK +. [file JBM4-6-e10670-s005.pdf]

# Supplementary Figure 3

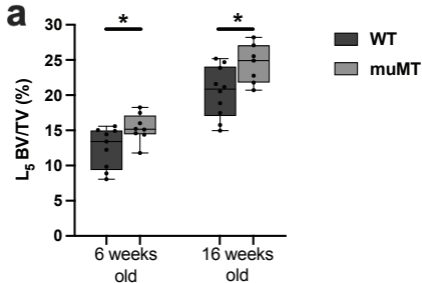

Supplement: Supplementary file 3 — Fig. S3 Female mice that lack mature B cells and immunoglobulins have increased trabecular bone in the axial skeleton. Trabecular bone volume per total volume (BV/TV) was analyzed with high‐resolution micro‐CT in vertebrae L5 in 6‐ and 16‐week old female mice. Student's t‐test was used to assess the differences between WT and muMT mice. N = 7–10. *p < 0.05. [file JBM4-6-e10670-s006.pdf]

# Supplementary Figure 4

**a**

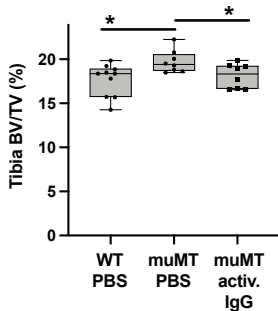

**b**

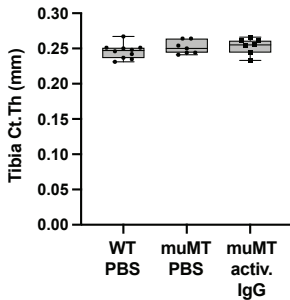

Supplement: Supplementary file 4 — Fig. S4 Polyclonal heat‐activated IgGs rescue the trabecular bone phenotype in muMT mice. (a) MuMT mice were intraarticularly injected in one hind leg with polyclonal heat‐activated IgG, while the contralateral leg was injected with PBS. WT mice with an intraarticular injection with PBS were used for comparison. Tibia trabecular bone volume per total volume (BV/TV) in the metaphysis and (b) cortical thickness (Ct.Th) in the diaphysis were analyzed with high‐resolution micro‐CT. N = 7–10. *p < 0.05 versus muMT + PBS, Student's t‐test was used to assess the differences. [file JBM4-6-e10670-s003.pdf]

# Supplementary Figure 5

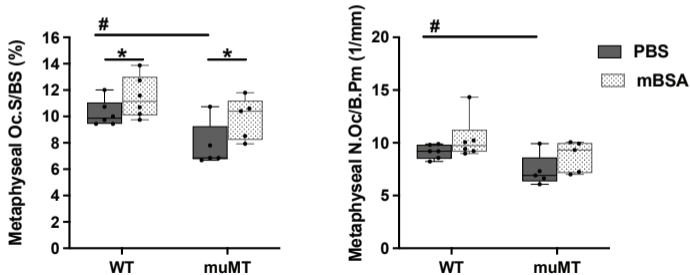

Supplement: Supplementary file 5 — Fig. S5 Osteoclast number and surface in metaphyseal bone in males. Male muMT and wild‐type (WT) littermate mice were immunized with methylated bovine serum albumin (mBSA). Antigen challenge was repeated intra‐articularly after 7 days. Number of osteoclasts per bone perimeter (N.Oc/B.Pm) and osteoclast surface per bone surface (Oc.S/BS) in the metaphyseal part of the tibia. N = 5–6. Unpaired Student's t‐test was used between WT and muMT, #p < 0.05, and paired t‐test was used for comparisons within the same mouse (non‐arthritic versus arthritic side), *p < 0.05. [file JBM4-6-e10670-s002.pdf]
