# Supplementary material for: Reduction of Mature B Cells and Immunoglobulins Results in Increased Trabecular Bone
Source: JBMR Plus. 2022 Aug 30;6(9):e10670. doi: 10.1002/jbm4.10670 (PMC9465004; doi:10.1002/jbm4.10670)
Supplement: Supplementary file 6 — Table S1 Physiological Assessment of muMT Mice. Weights of organs in wild‐type (WT) and muMT mice. N = 7–12. Student's t‐test, WT versus muMT. g; gram, mg; milligram Table S2 Expression Pattern in Bone Marrow (BM) and Vertebral Bodies. RNA expression in BM and vertebral bodies in 16‐weeks old female and male wild‐type (WT) and muMT mice. WT mice (control) is set to 1, and expression in muMT mice is related to the WT littermates' controls and displayed as fold change. N = 9–12. # < 0.07, *p < 0.05, Student's t‐test. OPG, osteoprotegerin; RANK, receptor activator of nuclear factor kappa‐B; RANKL, RANK‐ligand; CSFR, colony stimulating factor receptor; IL, interleukin; TNFα, tumor necrosis factor alpha; RoRC, RAR‐related orphan receptor C; NFATc1, nuclear factor of activating T cells 1; Cxcr1, chemokine C‐X2‐C motif receptor 1; NFκB, nuclear factor kappa‐B; TRAP, tartrate resistant acid phosphatase; ALP, alkaline phosphatase. Table S3 Expression Pattern of Osteoblast‐Associated Genes. Ex vivo stimulated osteoblasts from the appendicular skeleton from wild‐type (WT) and muMT mice. WT mice (control) are set to 1, and the expression in muMT mice is related to the control group and displayed as fold change. N = 4–5. Col1α1, collagen type 1 alpha 1; Runx2, runt‐related transcription factor 2; ALP, alkaline phosphatase. [file JBM4-6-e10670-s001.docx]

**Table I; Expression pattern in bone marrow (BM) and vertebral bodies**

|  | WT | muMT |
| --- | --- | --- |
| **BM** | | |
| **OPG** | 1.00±0.66 | 0.44±0.17* |
| **RANKL** | 1.00±0.40 | 2.29±0.29* |
| **RANKL/OPG** | 1.00±0.41 | 14.13±6.21* |
| **CSFR** | 1.00±0.17 | 1.26±0.14 |
| **IL-6** | 1.00±0.32 | 0.89±0.03 |
| **IL-17** | 1.00±0.03 | 0.79±0.06* |
| **IL-23** | 1.00±0.05 | 1.04±0.08 |
| **TNFα** | 1.00±0.29 | 0.81±0.11 |
| **RorC** | 1.00±0.17 | 1.33±0.14 |
| **NFATc1** | 1.00±0.10 | 1.04±0.16 |
| **Cx3Cr1** | 1.00±0.12 | 0.96±0.14 |
| **Vertebral bodies** | | |
| **OPG** | 1.00±0.08 | 0.67±0.07* |
| **RANKL** | 1.00±0.11 | 1.33±0.10# |
| **RANKL/OPG** | 1.00±0.10 | 1.70±0.19* |
| **RANK** | 1.00±0.06 | 0.93±0.07 |
| **NFATc1** | 1.00±0.22 | 0.40±0.09* |
| **TRAP** | 1.00±0.10 | 0.75±0.05* |
| **ALP** | 1.00±0.18 | 1.08±0.08 |

RNA expression in BM and vertebral bodies in 6 weeks old female wild-type (WT) and muMT mice. WT mice (control) are set to 1, and expression in muMT mice is related to the WT littermates’ controls and displayed as fold change. N=8-9. *#=0.07, *p<0.05*, Student’s t-test. *OPG*; osteoprotegerin, *RANK*; receptor activator of nuclear factor kappa-B, *RANKL*; RANK-ligand, *CSFR*; Colony stimulating factor receptor, *IL*; interleukin, *TNFα;* tumor necrosis factor alpha, *RoRC*; RAR-related orphan receptor C, *NFATc1*; nuclear factor of activating T cells 1, *Cxcr1;* chemokine C-X2-C motif receptor 1, *NFκB*; nuclear factor kappa-B, *TRAP*; tartrate resistant acid phosphatase, *ALP*; alkaline phosphatase.

**Supplementary Table I; Physiological assessment of muMT mice**

|  | WT | muMT | WT | muMT |
| --- | --- | --- | --- | --- |
|  | 6-week-old female | | 16-week-old female | |
| **Liver (g)** | 0.83± 0.02 | 0.75 ±0.03 | 0.96 ±0.06 | 0.85 ±0.06 |
| **Thymus (mg)** | 80.6 ±3.29 | 87.0 ±9.69 | 62.9 ±4.81 | 63.9 ±3.72 |
| **Uterus (mg)** | 63.7±17.5 | 44.7±12.8 | 78.6 ±9.6 | 70.6 ±8.8 |
| **Gonadal fat (mg)** | 87.5±14.7 | 89.0±12.8 | 289.5±65.0 | 230.8±50.1 |

Weights of organs in wild-type (WT) and muMT mice. N=7-12. Student’s t-test, WT vs muMT. g; gram, mg; milligram

**Supplementary Table II; Expression pattern in bone marrow (BM) and vertebral bodies**

|  | WT | muMT | WT | muMT |
| --- | --- | --- | --- | --- |
|  | Female | | Male | |
| **BM** | | | | |
| **OPG** | 1.00±0.21 | 0.56±0.18* | 1.00±0.16 | 0.41±0.16* |
| **RANKL** | 1.00±0.40 | 2.19±0.35* | 1.00±0.30 | 2.17±0.23* |
| **RANKL/OPG** | 1.00±0.41 | 11.03±4.51* | 1.00±0.81 | 7.90±2.68* |
| **CSFR** | 1.00±0.21 | 1.18±0.38 | 1.00±0.20 | 1.35±0.15 |
| **IL-6** | 1.00±0.22 | 0.90±0.09 | 1.00±0.08 | 1.01±0.09 |
| **IL-17** | 1.00±0.08 | 0.81±0.11* | 1.00±0.10 | 0.68±0.12* |
| **IL-23** | 1.00±0.35 | 1.35±0.35 | 1.00±0.28 | 1.21±0.35 |
| **TNFα** | 1.00±0.29 | 1.03±0.16 | 1.00±0.29 | 0.81±0.10 |
| **RorC** | 1.00±0.14 | 1.38±0.29 | 1.00±0.14 | 1.43±0.30 |
| **NFATc1** | 1.00±0.13 | 1.01±0.11 | 1.00±0.09 | 0.91±0.12 |
| **Cx3Cr1** | 1.00±0.22 | 1.36±0.30 | 1.00±0.18 | 0.97±0.13 |
| **Vertebral bodies** | | | | |
| **OPG** | 1.00±0.10 | 0.58±0.11* | 1.00±0.14 | 0.68±0.04* |
| **RANKL** | 1.00±0.12 | 1.29±0.18 | 1.00±0.07 | 1.24±0.06# |
| **RANKL/OPG** | 1.00±0.17 | 2.29±0.52# | 1.00±0.16 | 1.52±0.12* |
| **RANK** | 1.00±0.09 | 1.28±0.28 | 1.00±0.08 | 1.05±0.05 |
| **NFATc1** | 1.00±0.22 | 0.36±0.08* | 1.00±0.17 | 0.47±0.12* |
| **TRAP** | 1.00±0.08 | 0.79±0.09# | 1.00±0.10 | 0.75±0.05* |
| **ALP** | 1.00±0.14 | 0.85±0.08 | 1.00±0.18 | 0.90±0.07 |

RNA expression in BM and vertebral bodies in 16-weeks old female and male wildtype (WT) and muMT mice. WT mice (control) is set to 1, and expression in muMT mice is related to the WT littermates’ controls and displayed as fold change. N=9-12. *#<0.07, *p<0.05*, Student’s t-*OPG*; osteoprotegerin, *RANK*; receptor activator of nuclear factor kappa-B, *RANKL*; RANK-ligand, *CSFR*; Colony stimulating factor receptor, *IL*; interleukin, *TNFα;* tumor necrosis factor alpha, *RoRC*; RAR-related orphan receptor C, *NFATc1*; nuclear factor of activating T cells 1, *Cxcr1;* chemokine C-X2-C motif receptor 1, *NFκB*; nuclear factor kappa-B, *TRAP*; tartrate resistant acid phosphatase, *ALP*; alkaline phosphatase.

**Supplementary Table III; Expression pattern of osteoblast-associated genes.**

|  | WT | muMT |
| --- | --- | --- |
| **Osteocalcin** | 1.00±0.03 | 0.74±0.11 |
| **Col1α1** | 1.00±0.09 | 1.19±0.17 |
| **Runx2** | 1.00±0.02 | 0.87±0.08 |
| **ALP** | 1.00±0.13 | 1.01±0.13 |

*Ex vivo* stimulated osteoblasts from the appendicular skeleton from wild-type (WT) and muMT mice. WT mice (control) are set to 1, and the expression in muMT mice is related to the control group and displayed as fold change. N=4-5. *Col1α1*; collagen type 1 alpha 1, *Runx2*; runt-related transcription factor 2, *ALP*; alkaline phosphatase.
